# Temporal profiling of cytokines in passively expressed sweat for detection of infection using wearable device

**DOI:** 10.1002/btm2.10220

**Published:** 2021-04-01

**Authors:** Badrinath Jagannath, Kai‐Chun Lin, Madhavi Pali, Devangsingh Sankhala, Sriram Muthukumar, Shalini Prasad

**Affiliations:** ^1^ Department of Bioengineering University of Texas at Dallas Richardson Texas USA; ^2^ Department of Electrical Engineering University of Texas at Dallas Richardson Texas USA; ^3^ EnLiSense LLC Allen Texas USA

**Keywords:** cytokine biomarkers, infection, multiplexed detection, portable electronics, sweat, wearables

## Abstract

This work presents the viability of passive eccrine sweat as a functional biofluid toward tracking the human body's inflammatory response. Cytokines are biomarkers that orchestrate the manifestation and progression of an infection/inflammatory event. Hence, noninvasive, real‐time monitoring of cytokines can be pivotal in assessing the progression of infection/inflammatory event, which may be feasible through monitoring of host immune markers in eccrine sweat. This work is the first experimental proof demonstrating the ability to detect inflammation/infection such as fever, FLU directly from passively expressed sweat in human subjects using a wearable “SWEATSENSER” device. The developed SWEATSENSER device demonstrates stable, real‐time monitoring of inflammatory cytokines in passive sweat. An accuracy of >90% and specificity >95% was achieved using SWEATSENSER for a panel of cytokines (interleukin‐6, interleukin‐8, interleukin‐10, and tumor necrosis factor‐α) over an analytical range of 0.2–200 pg mL^−1^. The SWEATSENSER demonstrated a correlation of Pearson's *r* > 0.98 for the study biomarkers in a cohort of 26 subjects when correlated with standard reference method. Comparable IL‐8 levels (2–15 pg mL^−1^) between systemic circulation (serum) and eccrine sweat through clinical studies in a cohort of 15 subjects, and the ability to distinguish healthy and sick (infection) cohort using inflammatory cytokines in sweat provides pioneering evidence of the SWEATSENSER technology for noninvasive tracking of host immune response biomarkers. Such a wearable device can offer significant strides in improving prognosis and provide personalized therapeutic treatment for several inflammatory/infectious diseases.

## 1 INTRODUCTION

**FIGURE 1:**
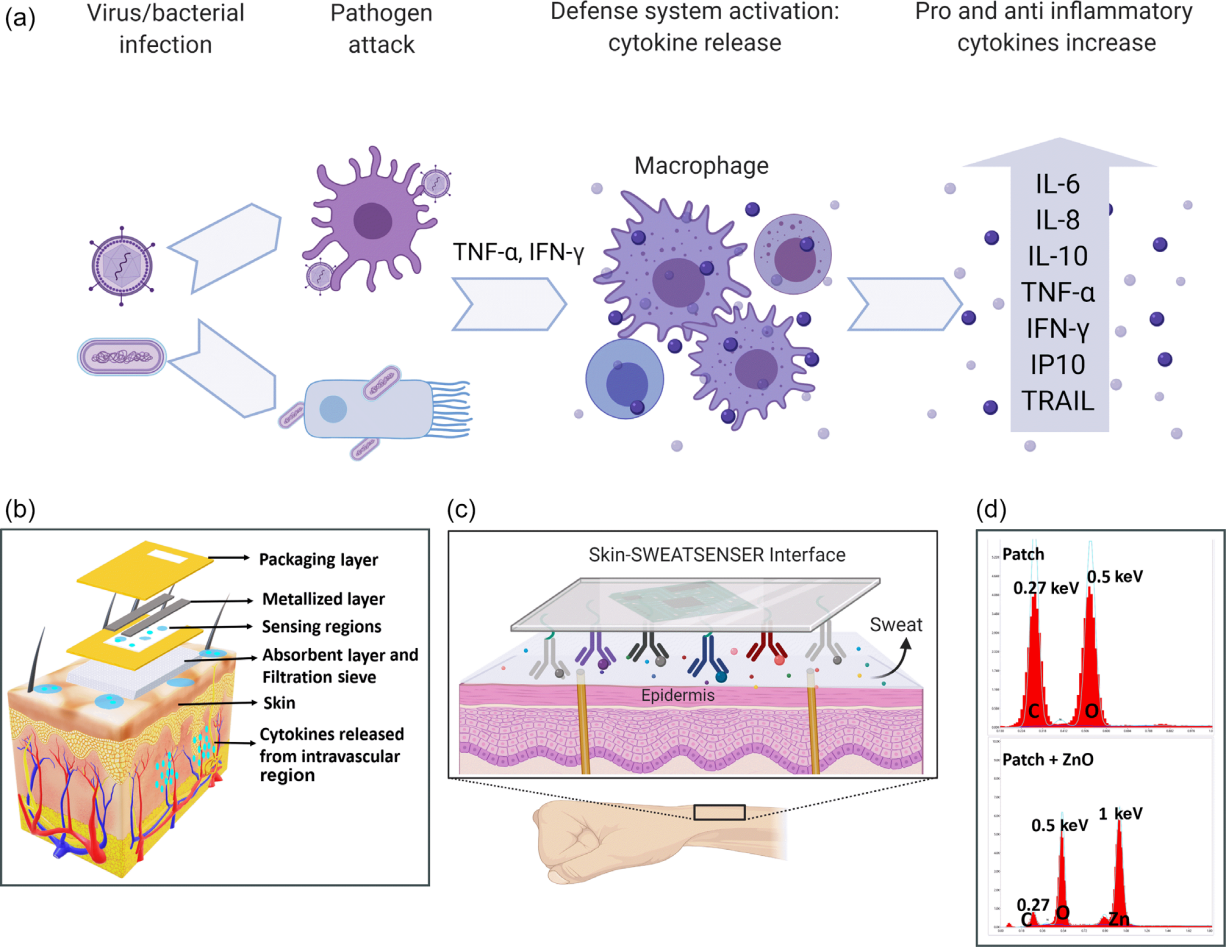
Schematics of immunological cascading effects of inflammatory response and SWEATSENSER device features. (a) Cytokine pathogenesis of the virus or bacterial infection. (b) Exploded view of layers of the SWEATSENSER strip on contact with skin. (c) Skin‐SWEATSENSER device interface, where SWEATSENSER is functionalized with specific antibodies to capture the study biomarkers. (d) EDX spectra of patch membrane and ZnO deposited on patch membrane. Carbon and oxygen peaks at 0.27 and 0.5 keV, respectively, are observed due to the polymeric structure of porous patch. Upon deposition of Zinc oxide, a peak at 1 keV corresponding to L‐shell of Zinc with an increase in oxygen peak intensity and decrease of carbon peak is observed. Figure (a–c) were created in Biorender

Cytokines are low molecular proteins produced by the immune system in response to external infectious agents or inflammatory event.^1^, ^2^ They play an important role in protecting against viral and bacterial infections^3^ and are actively involved in the pathogenesis of infection. Cytokines induce biological effects to act as powerful mediators of immune response to counteract infectious pathogen attack.^4^ In the event of an infection due to acute respiratory diseases such as influenza, COVID‐19, the complex interplay between the viral pathogen virulence and host resistance dictates the severity of progression of disease in the host.^5^ The complications associated with such infections are often attributed to hyperinduction of pro‐inflammatory cytokine production, and in severe cases leads to a phenomenon called as “cytokine storm.”^5^, ^6^, ^7^, ^8^ High levels of tumor necrosis factor (TNF), cytokines, and chemokines were determined in cytokine storm events pertinent to influenza and more recently with SARS‐CoV‐2 patients.^9^, ^10^, ^11^ Figure 1(a) illustrates a series of systemic events and mediators resulting in the release of several pro‐inflammatory and anti‐inflammatory cytokines such as tumor necrosis factor‐α (TNF‐α), interleukin‐6 (IL‐6), interleukin‐8 (IL‐8), and interleukin‐10 (IL‐10) due to a bacterial or viral pathogen attack.^12^ Cytokine levels, therefore, orchestrate the time course of infection progression in a host.^13^ Hence, tracking the cytokines associated with infections from the time of pathogen attack in the host can effectively be used for improved prognosis and better management of an infection. Real‐time monitoring of critical inflammatory cytokine markers will not only aid in early disease risk stratification and mitigation but can also help guide clinicians in customizing treatments based on host immune response.

However, there are no continuous monitoring systems that can report the levels of cytokines in real time as current methods rely on blood plasma/serum or saliva as the biofluid for detecting these markers, which is not suitable for continuous monitoring. In contrast, eccrine sweat as a biofluid is an excellent alternative for real‐time, non‐invasive monitoring of cytokines. Passively expressed eccrine sweat has a plethora of biological information that comprises of ions, small molecules, metabolites, and proteins.^14^, ^15^ Figure S1 (in supporting information) illustrates the mechanism of release of cytokines into sweat where each eccrine gland consists of a secretory coil and a dermal duct. Various chemical species (hormones, ions, metabolites, acids, small proteins, and peptides) enter the sweat through the dermal duct and secretory coil.^16^ During an inflammatory response, cytokines are released from the intravascular membrane. The adherent intercellular junctions get diminished, causing immunoglobulins, cytokines, and water to move into the dermal interstitium.^17^ These cytokines and other protein molecules then diffuse into eccrine sweat gland and reach the skin surface by hydrostatic pressure. Cizza et al.^18^ and Marques‐Deak et al.^19^ have quantified the levels of IL‐6, IL‐8, and TNF‐α in sweat. Furthermore, the levels of the cytokine markers between healthy cohort and major depressive disorder (MDD) patients were easily distinguishable. Additionally, Dai et al. validated the presence of IL‐10 in sweat.^20^ Although all these studies are significant in validating the presence of cytokines in sweat, they were single‐point measures that required cumbersome processing methods and then measured using standard laboratory techniques.

The advent of wearable technology‐enabled hardware and software platforms has allowed several researchers to demonstrate the feasibility of sensor technologies for continuous monitoring of biomarkers in sweat. A vast majority of them are focused on ions (sodium, potassium, chloride), metabolites, and small molecules (glucose, cortisol) from stimulated sweat. However, passively expressed sweat (i.e., sweat expressed without any external stimulation methods such as iontophoresis, exercise) is critical for measuring and correlating cytokines to that in circulation (serum). A stimulation event may be viewed as being synonymous to an inflammation event and therefore may not be an actual representation of the infection/inflammatory event driven cytokine levels in circulation. Further, other limitations such as limited duration of sampling and limited volume from stimulated sweat can impede monitoring of cytokines.^21^ Therefore, this work is the first report of demonstration of a wearable SWEATSENSER technology platform that measures cytokine biomarkers in passively expressed eccrine sweat in quadruplex manner to distinguish between healthy cohort and sick cohort. The sweat levels have been analytically correlated with blood serum cytokine levels.

The wearable SWEATSENSER device platform uses an electrochemical biosensor strip that is functionalized with capture probes specific to the study biomarkers and a reader for electrochemical transduction for real‐time, continuous reporting of cytokines in sweat. The schematic of the sensor strip stack is represented in Figure 1(b). The sensor strip comprises of multiple fluid transport sites that have been carefully designed to ensure effective capture of sweat. An absorbent layer of an FDA‐approved PharmChek patch interfaces with the skin to capture the sweat, and the sweat diffuses through the porous sieve that allows the biomolecules to diffuse. The next layer comprises sensing regions functionalized with specific capture probe antibodies via a cross‐linker on semiconducting zinc oxide (ZnO) nanofilm (100–200 nm in thickness) that entraps the target biomarkers. The electrochemical binding interactions are transduced through the metallized layer. The sweat then diffuses into the next layer to be released out. The top‐most layer is a packaging layer that allows for the used sweat to release out and prevent any external moisture to enter, thus maintaining the fluid dynamics of the sensor strip in a controlled manner. Figure 1(c) illustrates a schematic of the skin‐SWEATSENSER interface when the SWEATSENSER is in contact with skin. The device is designed to rapidly detect and track levels of biomarkers in a multiplexed manner in conjunction with the vitals of a person toward establishing progression of infection post‐exposure and prior to the manifestation of symptoms. The wearable device also simultaneously measures skin temperature and perspiration for assessing vitals and the user sweating profiles respectively. We analytically validated sweat cytokine levels with serum cytokine measurements. The developed SWEATSENSER can differentiate healthy cohort from sick subjects with fever or viral infection such as influenza. This critical finding provides pioneering evidence that reporting sweat cytokine levels in a continuous manner as demonstrated with the SWEATSENSER device platform can be used for designing noninvasive wearable diagnostics for infection monitoring. The SWEATSENSER device can be useful in monitoring the host‐immune states that is vital and is a characteristic of the “wellness” to “illness” and back to “wellness” state in the patient. Such a technology platform can welcome a radical change in point‐of‐care (PoC) diagnostics for monitoring infections during seasonal epidemics and pandemic diseases.

## 2 RESULTS AND DISCUSSION

### 2.1 Characterization of SWEATSENSER

We first performed material characterization of the SWEATSENSER using SEM and EDAX to ensure the zinc oxide nanofilm was uniformly deposited on the patch membrane. Figure 1(d) demonstrates the EDX spectrum of the patch membrane before and after ZnO deposition. The carbon and oxygen peaks at 0.27 and 0.5 keV of only patch membrane (Figure 1(d) [i]) correspond to the polymeric structure of the PharmChek patch. After deposition of ZnO on the patch membrane, a distinct peak observed at 1 keV corresponds to the L‐shell of zinc (Figure 1(d) [ii]) with an increased peak height of oxygen at 0.5 keV. The corresponding SEM image of uniform deposition of ZnO is demonstrated in Figure S2A (Supporting information). Next, the immobilization of the immunoassay owing to the binding of the capture probe via a thiol cross‐linker on the sensor surface was confirmed using Fourier‐transform infrared spectroscopy (FTIR) (Figure S2B, Supporting Information). The top spectrum indicates the binding of the cross‐linker on the sensor surface with NHS ester bond (peak at 1780 cm^−1^) and free carboxylic acid peak (1740 cm^−1^). The conjugation of both antibodies shows cleaving of C—O bond of NHS ester and the peak at 1780 cm^−1^ disappears, while enhanced aminolysis peak (at 1652 cm^−1^) is observed confirming the binding of the antibody. Furthermore, electrochemical characterization was also performed to confirm the functionalization of the antibody. Electrochemical impedance spectroscopy over a frequency range of 1 Hz to 1 MHz was first performed on a blank ZnO deposited sensor by drop casting phosphate‐buffered saline (PBS) solution without any antibody functionalization. As observed from Nyquist plot in Figure S3A (Supporting information), a high impedance was observed indicating no antibody functionalization. The impedance dropped drastically upon antibody functionalization due to the charge modulation at the electrode/solution interface by the antibodies (Figure S3B). It should be noted that the Nyquist plot has an incomplete circle as this is a non‐faradaic method. The electrical equivalent circuit of the interface is represented in Figure S3C. To further confirm the binding of the antibody on the sensor surface, the obtained Nyquist plots were fit to the equivalent circuit. It can be observed from Figure S3D, the double layer capacitance (*C*_dl_) is very low for the blank ZnO sensor as there is no charge modulation occurring, and the low *C*_dl_ is contributed by the ions in the buffer solution. This result corroborates well with Figure S3A, where the impedance is very high. On the contrary, for the antibody functionalized sensors, a higher capacitance is observed owing to charge modulation by the bound antibody on the sensor surface. It is also observed that different antibodies exhibit varying double layer capacitance, indicating that the charge complex of each antibody is different, thus confirming the binding of antibody on the sensor surface.

**FIGURE 2:**
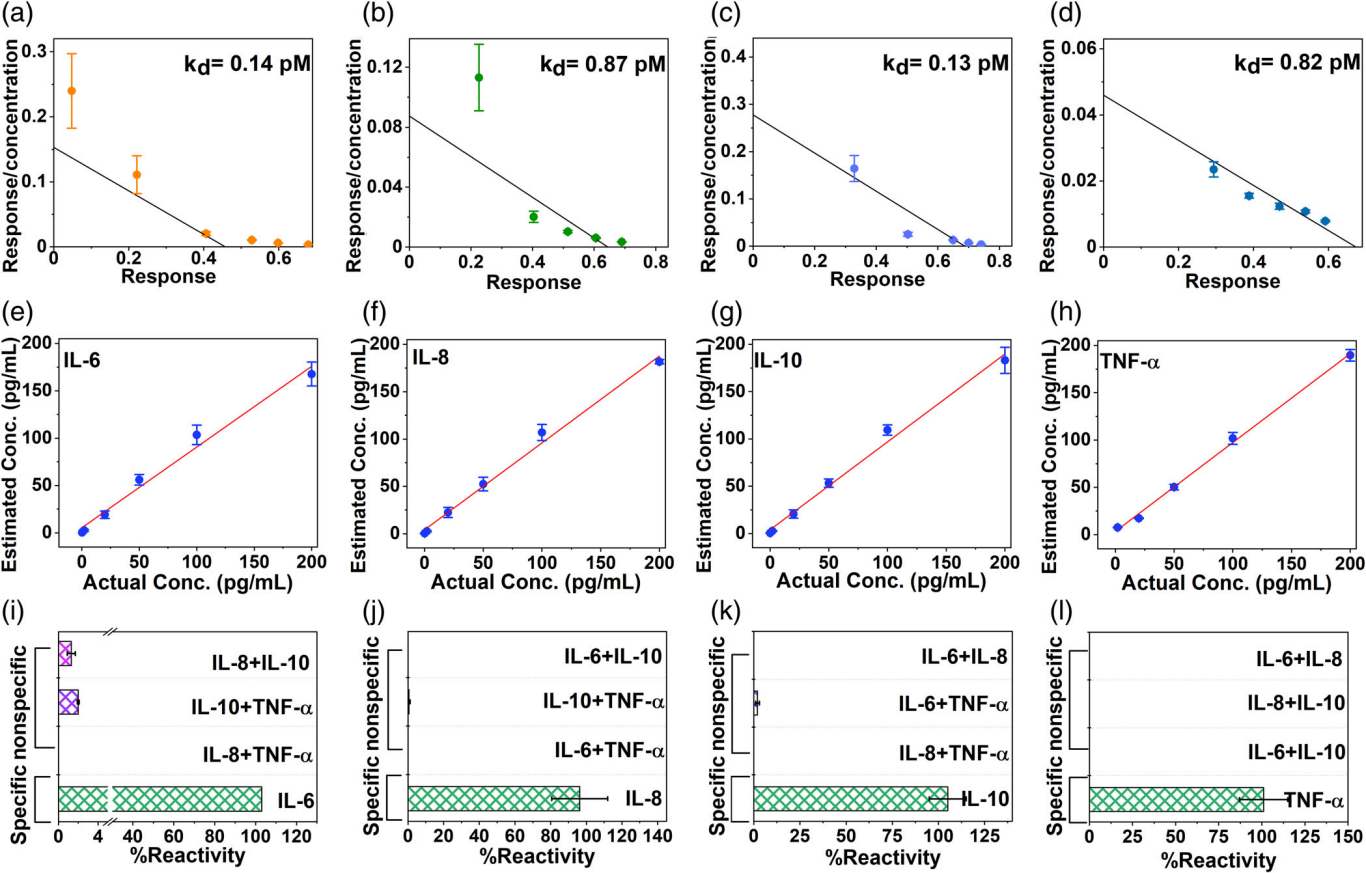
Characterization of SWEATSENSER. (a–d) Binding efficiency determined through *K*_d_ values for cytokines (a, IL‐6; b, IL‐8; c, IL‐10; d, TNF‐a). (e–h), Spike and recovery plots demonstrating ability to report concentrations reliably for cytokines in sweat (e, IL‐6; f, IL‐8; g, IL‐10; h, TNF‐a). Linear regression analysis was performed between “Actual” and “Reported” concentration and *R* ^2^>0.95 was achieved for *n* ≥ 3 measures. (i–l) Selectivity and specificity of SWEATSENSER for target cytokine marker in sweat (i, IL‐6; j, IL‐8; k, IL‐10; l, TNF‐a); SWEATSENSER demonstrates minimal (<2%) or no response to nonspecific molecules, while it is highly specific (>97%) to target biomarker. Data are represented as mean ± SEM

After validating the successful immobilization of the capture probe antibody on the sensor surface, the immune‐sensing performance of the SWEATSENSER was evaluated to understand the binding affinity between the capture probe and target molecule as the developed sensing system works on an affinity‐based mechanism. Firstly, the affinity of the capture probe antibodies to the target analyte was determined using the saturation binding curve studies by measuring the equilibrium constant (*K*_d_). Typically, *K*_d_ is used as a key parameter in determining the ability of the antibody to quickly dissociate and capture the antigen molecule. Concentrations of the analyte were varied and the impedimetric response was measured to determine the *K*_d_ for each capture probe using saturation binding curve‐fitting equations in Graphpad Prism as described in Supporting Information. Affinity of an antibody is inversely proportional to the *K*_d_. Ideally, an excellent affinity‐based system will have a *K*_d_ value in the lower picomolar regime for a cytokine affinity capture probe.^22^ The SWEATSENSER satisfies this criterion with a very low *K*_d_ of 0.14, 0.83, 0.13, and 0.82 pM for IL‐6, IL‐8, IL‐10, and TNF‐α, respectively (Figure 2(a–d)) for *n* = 3 measures, thus indicating high affinity of the capture probe antibody used for target marker detection. Such low equilibrium constant also reflects high sensitivity for detecting ultra‐low analyte concentrations, which is important specifically for sweat‐based systems. After establishing high affinity of the capture probes to the target markers in sweat, we evaluated whether the SWEATSENSER can report the levels of the study markers reliably. In order to determine this, SWEATSENSER was functionalized with specific capture probe antibody individually to measure the response devoid of crosstalk. A series of six concentrations ranging from 0.2 to 200 pg mL^−1^ (in the physiological range) for each study marker were prepared independently in neat sweat (without nonspecific molecules) and dispensed on the corresponding sensor. Electrochemical impedance spectroscopy (EIS), the core sensing technology (described in the Methods section), of the SWEATSENSER was used to record the signal response and the corresponding concentrations were estimated. Nyquist plot for varying concentrations is demonstrated in Figure S4 (Supporting Information). Non‐faradaic EIS is a powerful technique that can measure subtle changes in the electrical double layer due to binding interactions between capture probe–target analyte without the need for redox labels by application of a small input AC voltage.^23^ The measured impedance associated with target biomolecule binding is a complex value, since the current response can differ in terms of not only the amplitude but can also show a phase shift (φ) with respect to the voltage–time function. Therefore, the results of an impedance measurement can be illustrated using a bode plot (which plots absolute impedance (|*Z*mod|) and phase (φ) as a function of frequency).^24^ The resulting response was calibrated to a fixed frequency for reporting the concentration values of the target in the sample specimen. All this computation and analysis were performed using the handheld electronic reader onto which the sensors are mounted, and the output results of concentration over time can be interfaced via Bluetooth to report on a smartphone app. EIS can effectively differentiate the binding interactions from any surface and bulk effects such as pH and conductivity variations through frequency tuning and thus, providing a molecular specific response. These bulk effects occur beyond the electrical double layer and are a characteristic at high frequency, whereas, the binding interactions occur in the low frequency regime of <1000 Hz.^25^, ^26^, ^27^ Therefore, the response to the specific interaction was captured at 180 Hz. It is observed from Figure 2(e–h) that the SWEATSENSER reliably determined the levels of the study biomarkers. Further, SWEATSENSER demonstrated a linear response with *R* ^2^ > 0.98 over a 3 log‐order dynamic range of 0.2–200 pg mL^−1^ for all the study markers of interest for at least *n* = 3 measures. This dynamic range conveniently covers the range for determining infection levels including acute infections such as influenza, SARS‐CoV‐2, and sepsis.^28^ SWEATSENSER was further evaluated for its selectivity and specificity to the study biomarkers. The sensors were prepared in a similar manner as the previous experimental design reported for linearity. Three combinations of cocktail solutions were prepared by mixing two non‐specific analytes in sweat analog at a high concentration of 200 pg mL^−1^ (final concentration after mixing) to determine the cross‐reactivity of each capture probe with the nonspecific molecules. This solution was devoid of the specific target biomolecule. The prepared cocktail of nonspecific markers was dispensed on the sensor and the response was recorded. The reactivity was determined as a percentage ratio of measured concentration to the actual concentration of the biomarkers. The SWEATSENSER demonstrates excellent selectivity with negligible (<2%) or no reactivity to the non‐specific molecules while a high signal response (~> 98%) was reported for the specific marker as demonstrated in Figure 2(I‐L).

### 2.2 Evaluation of SWEATSENSER analytical performance metrics

**FIGURE 3:**
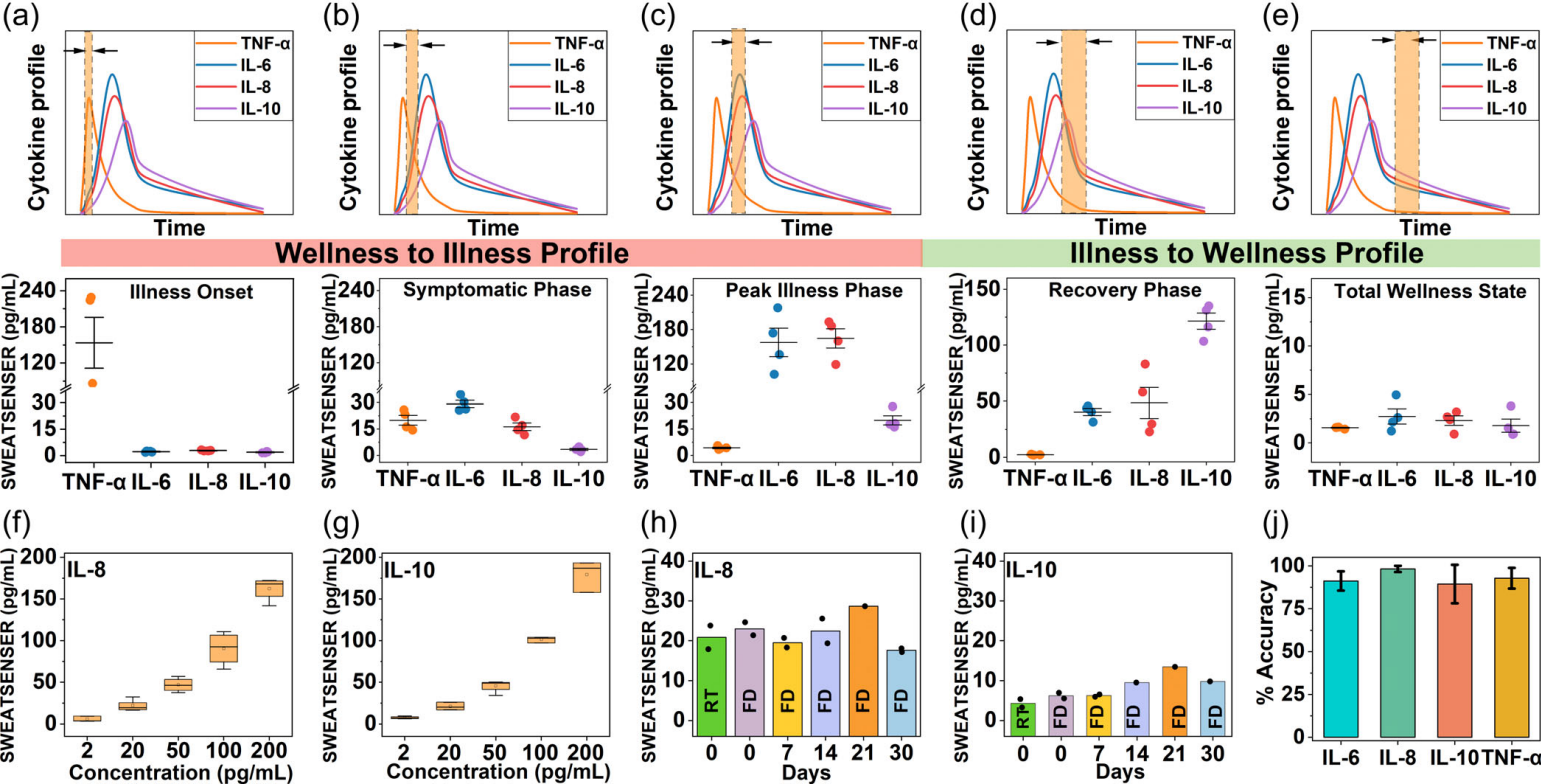
Analytical metrics of SWEATSENSER. (a–e) Ability of SWEATSENSER to reliably track biomarker levels during various stages of infection from early‐stage pathogen attack to recovery. Temporal profiles of biomarkers during various stages of infection (top part) and the corresponding levels reported by SWEATSENSER are represented as whisker plot. The band with double‐sided arrow (indicated in the top half of the graph) indicates time snapshot of cytokine profile during various stages of infection. Specifically, (a,c) demonstrate the SWEATSENSER response tracking of cytokine markers during wellness to illness phase and (d,e) demonstrate the recovery phase, which is illness to wellness. (f,g) Reproducibility of SWEATSENSER demonstrated with pro‐inflammatory (f, IL‐8) and anti‐inflammatory (g, IL‐10) cytokines for *n* ≥ 4 sensors. (h,i) Time stability of SWEATSENSER for (h, IL‐8) and (i, IL‐10) cytokines. RT refers to sensor incubated in room temperature without lyophilization and FD refers to freeze‐dry (i.e., lyophilized sensor) for *n* = 2 measures. (j) Accuracy of SWEATSENSER in reporting cytokine markers in sweat. Data are represented as mean ± SEM. Lyophilized SWEATSENSERs were used for reproducibility and stability studies

The temporal profile of cytokines orchestrates the time course of an infection event and recovery from infection.^29^ Dynamic profiling of host immune markers using infection monitoring wearable devices can significantly mitigate the effects of a pathogen attack by aiding in presymptomatic alarm and early therapeutic intervention. Therefore, the study presented in this section evaluated the potential of the SWEATSENSER to be synergistically used as a wellness dashboard for reporting the temporal dynamic changes in levels of the study biomarkers. We evaluated the functionality and the response of the SWEATSENSER by mimicking the temporal profile of the host immune cytokine markers in response to the infectious pathogen attack as represented in Figure 3(a–e). The band (in orange with double‐headed arrow) in the cytokine profile graphs indicate the time snapshot window of the body's immune host biomarker response due to an infection trigger. This snapshot reveals the relative biomarker levels at a specific time interval during various stages of an infection/inflammatory event. To assess the ability of the SWEATSENSER to report the levels reliably in a multiplexed manner, the temporal infection profile was classified into five stages right from onset to recovery. In this context, Figure 3(a–c) describes the cytokine profile of the wellness to illness phase, that is, from infection onset to illness, while Figure 3(d,e) represents the temporal snapshot of illness to recovery phase. Based on the relative profile of the biomarkers, the sensor was dispensed with concentrations pertinent to that specific time interval and the reported levels were calculated for *n* = 4 measures per biomarker at each inflammatory response phases. In an infectious event, the pro‐inflammatory response of TNF‐α increases rapidly that triggers other pro‐inflammatory markers such as IL‐6 and IL‐8.^30^ As the levels of IL‐6 and IL‐8 elevate and peak, the anti‐inflammatory markers such as IL‐10 begin to elevate to help the body in immuno‐suppression.^31^ As seen from Figure 3(a–e), the SWEATSENSER can reliably report the relative temporal levels of cytokine markers during various stages of inflammatory response, thus being suitable for dynamic profiling in the event of an infection. As the temporal profile of the study markers change dynamically during an infection, it is critical for a continuous monitoring system to be stable, long‐lasting, and reliable without being prone to drifts due to external and environmental factors. Hence, we investigated the SWEATSENSER through critical evaluation of performance metrics such as reproducibility, precision, accuracy, and stability.

The performance of biosensors that function based on immunoassay mechanism is primarily dependent on the stability and specificity of the capture probe. The sensor strips were functionalized with specific antibodies and then lyophilized to enhance the shelf‐stability. Lyophilization removes water and prevents the antibody from denaturing.^32^ The samples were then vacuum sealed and opened just prior to testing the sensors. Firstly, the ability to demonstrate consistent response with different sensors was validated using reproducibility studies. Reproducibility determines the degree of closeness in response between different sensors comprehending the analytical range of the study biomarker. Here, five different sensors for each of pro‐inflammatory (IL‐8) and anti‐inflammatory (IL‐10) were measured. The sensors were dosed with concentrations from 2 to 200 pg mL^−1^, and the corresponding reported concentration was calculated from the calibration curve to determine if the response is identical from five sensors. Figure 3(f,g) represents the box plots for reproducibility response of pro and anti‐inflammatory cytokines. It is observed that the SWEATSENSER demonstrates a reproducible response between multiple sensors over the entire range of 2 to 200 pg mL^−1^. Additionally, the nonoverlapping interquartile ranges of the box plots confirm the ability of the sensor to reliably distinguish concentration levels of the cytokines. Figure S5 (Supporting Information) demonstrates the precision of the sensor indicated as %CV (coefficient of variation) for *n* = 4 measurements. The SWEATSENSER reported a CV < 10% for all the study markers, which is well below the Clinical and Laboratory Standards Institute (CLSI) standards of an acceptable CV below 20%.^33^

Stability of the wearable sensors is also critical for practical use on human subjects. Therefore, we evaluated whether the stability of the SWEATSENSER is retained at least for a month without loss in sensitivity. Five batches of sensors were functionalized each with pro‐inflammatory (IL‐8) and anti‐inflammatory (IL‐10) capture probe antibodies. The functionalized sensors were then lyophilized and immediately vacuum sealed for storage. The response of the lyophilized sensors was evaluated by dosing ~20 pg mL^−1^ IL‐8 and ~5 pg mL^−1^ IL‐10 on the corresponding specific antibody functionalized sensors. The stability of the SWEATSENSER platform was assessed by measuring the response of the sensors on Day 0, 7, 14, 21, and 30 from the day of the antibody functionalization and lyophilization. Figure 3(h,i) demonstrates that the stability of the sensor is retained over a month without a loss in signal response for *n* = 2 measures on each day of measurement. The SWEATSENSER reported a concentration ~18–24 pg mL^−1^ for IL‐8 concentration of 20 pg mL^−1^ and ~5–10 pg mL^−1^ for the IL‐10‐dosed concentration of 5 pg mL^−1^, indicating that the sensitivity is retained for 1 month even at the low concentration regime. Furthermore, the lyophilized sensors (indicated as FD in bar plots) demonstrate similar response to that of the sensors measured directly after functionalization without lyophilization (indicated as RT in bar plots), thus confirming reliability of SWEATSENSER for over month. Mechanical resiliency of the SWEATSENSER was determined by subjecting to repeated stress, strain cycles through hand movements as in the real‐case scenarios (Figure S6, Supporting Information). SWEATSENSER demonstrated stable response even after 100 cycles of repeated mechanical motion (Figure S6, Supporting Information), with no loss in signal response, signifying the mechanical stability of the device. The SWEATSENSER reported an accuracy >90% for all the study biomarkers (Figure 3j). Thus, these detailed and rigorous characterization results elucidate the robustness and signify operability of the SWEATSENSER for real use‐case scenarios.

### 2.3 Pre‐clinical utility of SWEATSENSER on healthy subjects

**FIGURE 4:**
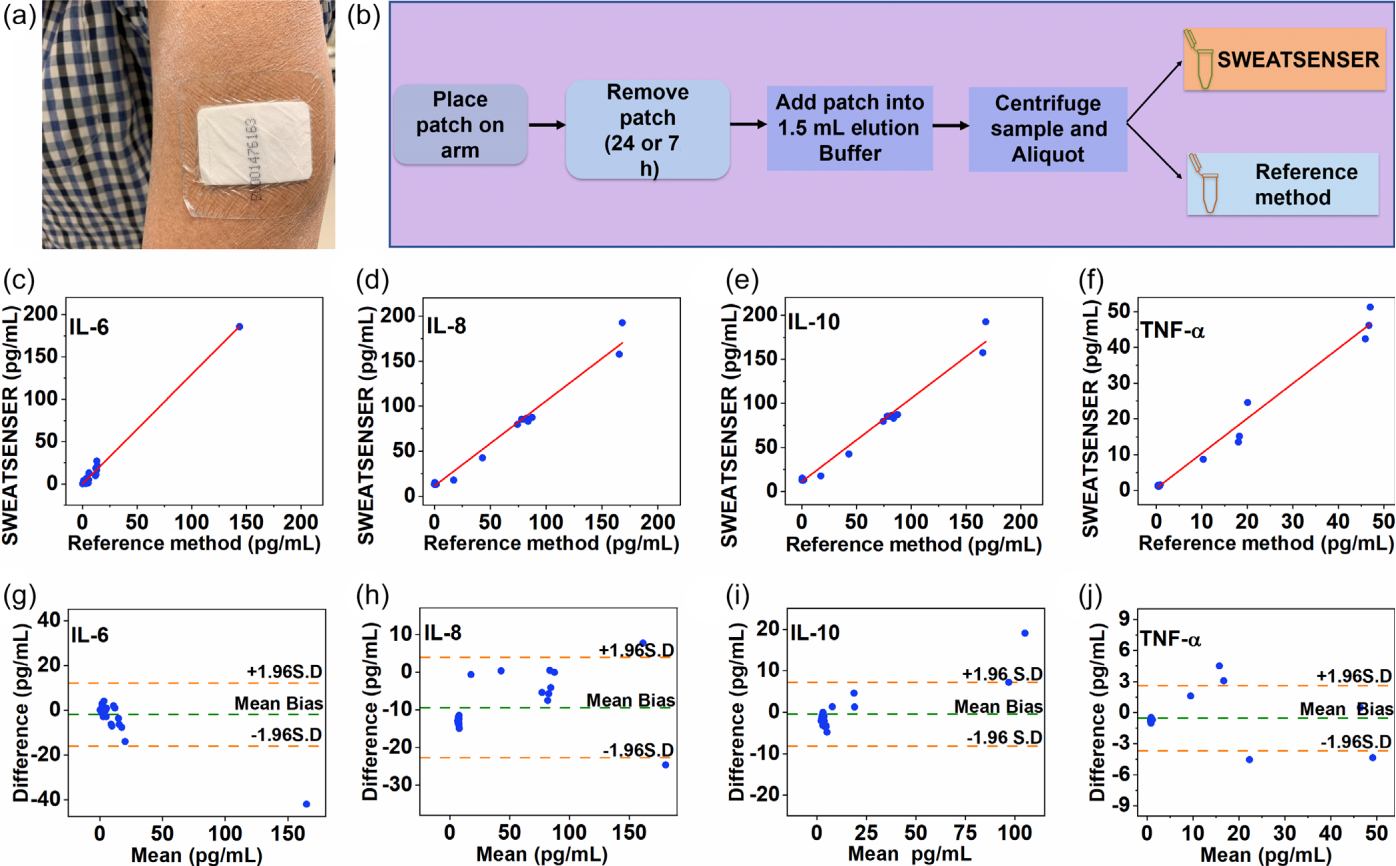
Validation of SWEATSENSER with standard reference method for human subject samples. (A) PharmChek sweat patch worn by subject on arm. (b) Schematic of sweat sample collection using patch for correlation of SWEATSENSER with ELISA. (c–f) Pearson's correlation between SWEATSENSER and reference ELISA method with Pearson's correlation coefficient *r* = 0.99 for all biomarkers from *n* = 26 healthy human subject measures. (g–j) Bland–Altman analysis between SWEATSENSER and ELISA from *n* = 26 healthy human subject measures with a mean bias −1.99 pg mL^−1^ IL‐6 (g), −9.43 pg mL^−1^ IL‐8 (h), −0.5 pg mL^−1^ IL‐10 (i), and 0.55 pg mL^−1^ TNF‐α (j)

In order to use the developed SWEATSENSER for clinical use‐cases, it is imperative for the device to demonstrate a good agreement with the current standard reference methods. Therefore, we compared whether the SWEATSENSER performance is congruent to standard commercial ELISA. Sweat was passively collected using an FDA‐approved sweat collection PharmChek® patch from 26 healthy volunteers who had no reported signs or symptoms of infection in compliance with the protocol approved by IRB. PharmChek® patches have been widely used for the analysis of several molecules including proteins and cytokines in sweat.^34^, ^35^, ^36^, ^37^ The protocol for sample collection, processing, and handling was adapted from Hladek et al.^37^ Figure 4(a) represents passive eccrine sweat collection from the arm of volunteers, which is a high sweat gland density region ~110–120 glands/cm^2^.^38^ Table S1 in Supporting Information summarizes the healthy cohort information. After collecting and processing the sample as illustrated in the schematic in Figure 4(b), the samples were tested on both the SWEATSENSER and ELISA. Pearson's correlation and Bland–Altman analysis were implemented to determine the degree of agreement between SWEATSENSER and standard reference method. The two methods show excellent linear correlation with Pearson's coefficient *r* = 0.99 for all the study markers (Figure 4(c–f)). Additionally, Bland–Altman analyses confirm agreement between the results using two methods of detecting the biomarkers. A very low mean bias of −1.99, −9.43, −0.5, and 0.55 pg mL^−1^ was achieved for IL‐6, IL‐8, IL‐10, and TNF‐α, respectively (Figure 4(g–j)). Although the mean bias is higher for IL‐8, it is well within the limits to clearly differentiate healthy and sick levels. All the sample points are scattered around the mean bias line indicating that neither of the methods overpredict or underpredict. The tight 95% CI bands (±1.96 SD) further confirm that there is no significant deviation between SWEATSENSER and reference method. A continuous monitoring sweat analytics platform involves a dynamic accumulation of sweat on the patch; therefore, we wanted to understand if this phenomenon would alter or cause any variation in the marker levels over time. Two patches, one on each arm, were put on some of the healthy subjects for this investigation. One patch was removed at 24 h while the other was removed at 72 h. We found that the levels do not vary significantly between 24 and 72 h with a statistical insignificance *p* > 0.05 (95% CI), indicating that sweat biomarker levels do not alter with time in healthy cohort (Figure S7, Supporting Information). These results affirm that the temporal levels in healthy cohort do not significantly vary over time and dynamic change in the levels may imply an infection/inflammatory trigger.

### 2.4 On‐body testing of cytokines in passive eccrine sweat

Although there has been significant amount of work on continuous monitoring in sweat, most of it has been on the detection ions, metabolites, and small molecules such as Na^+^, K^+^, lactate, glucose, and cortisol^39^, ^40^, ^41^ through external stimulation of sweat. Only recently, Alexander et al.^42^ and Jagannath et al.^43^ have demonstrated detection of cytokines such as IL‐1α, IL‐1Ra, and IL‐1β collected in a patch for wearable applications from passive sweat. However, these were performed only on healthy cohort and did not involve any serum to sweat assessment. We believe that passive sweat collection for real‐time detection would offer significant strides in sweat‐sensing wearable technologies. The two most common practices for continuous monitoring of sweat biomarkers are exercise and stimulation by iontophoresis. Although sweat analytics through exercise may be useful for certain activity‐based applications, it is not a viable option for monitoring and tracking biomarkers in infectious patients. The other method, iontophoresis, involves stimulation of sweat glands. This method can cause inflammation and discomfort,^44^ which may lead to inaccurate cytokine levels influenced by localized inflammation of stimulated site rather than actual inflammatory response of infection. Moreover, sweat can be induced only for a limited time^45^ and requires periodic stimulation, which may not be suitable for implementing on infectious patients. Other limitations with iontophoresis include limited sweat volume and suppression of markers in induced sweat.^46^ Furthermore, we identified that biomarkers such as IL‐6 and TNF‐α were not readily expressed in induced sweat, whereas passive sweat collection showed the presence of all the study biomarkers (Table S3, Supporting Information). Considering these, it is imperative to have a sweat sensing system that relies on passive collection of the biofluid for monitoring infection markers.^44^ Thus, the SWEATSENSER was evaluated for on‐body monitoring of biomarkers from passively perspired sweat. First, three healthy subjects were recruited to understand the cytokine profile over time. Participants were refrained from any rigorous activity such as exercise throughout the duration of test to ensure the sweat was collected in a passive manner.

The SWEATSENSER device was placed on the lower arm that has high sweat gland density of 130–134 glands/cm^2^.^38^ SWEATSENSER has an effective area of 1.34 cm^2^ and has been designed such that it can report levels reliably even with low sweating rates. Typically, the sweating rate can be ~1–3 nL gland^−1^ min^−1^.^14^ Therefore, the sweating volume can be estimated as follows:

(1) $$\text{Sweating rate}\;\left(\vartheta \right)=\rho *\text{gland density}*A,$$

where *ρ* is the sweat rate per gland (μL min^−1^), *A* is the effective sweating area (cm^2^).

The effective sweating volume would amount to ~0.2–0.6 μL min^−1^ computed from the abovementioned equation. The device also houses a temperature and perspiration (reported in % relative humidity [%RH]) sensor. The purpose of the temperature sensor is two‐fold; one to measure the skin temperature to ensure that device was in contact with the skin and the other to confirm body vital temperature to assess whether the person has fever. The RH sensor was used to gauge the relative sweating profile of the subject (Figure S8 in supporting information).

As the developed sensor is based on an affinity capture assay method, the number of binding sites may decrease over time during continuous detection. Therefore, the sensor surface was sufficiently functionalized with the capture probe to ensure that sensor does not saturate for the measured time duration as previously demonstrated.^47^ Furthermore, we devised a unique data analytics strategy through implementation of an analytical framework to report concentrations in real time (Figure S9, Supporting Information). The developed analytical algorithm considers absolute impedance, change in impedance, and running difference in impedance to assess the levels as described in detail in Supporting Information. Impedance measurements were recorded for every 1 h of target analyte binding on the sensor surface. Changes in cytokine levels may dynamically change over a few hours; therefore, we hypothesize that such a sampling rate will provide a temporal snapshot of cytokine profile. Initial baseline measurement was performed. The previously measured impedance parameters such as running difference and normalized change in impedance were calculated. This was to account for the binding sites occupied on the sensing surface until the previous measurement step and recalibrate the sensor with respect to the previous step. All the abovementioned signal responses were used as features to train the ensemble model to achieve high accuracy with *R* ^2^ = 0.98 and low root mean square error (RMSE) = 7.8 (Table S4, Supporting Information). Using this developed analytical framework, the levels of the biomarkers were determined for continuous on‐body measurements.

Figure S10(A‐C) in supporting information demonstrates the cytokine concentrations in three healthy subjects measured every 1 h for a total of 4 h. Their corresponding temperature and sweating profile (as %RH) are represented in Figure S8. It is observed in Figure S10 that the levels are <12 pg mL^−1^ for IL‐6, IL‐8 and IL‐10. Although a slight increase was observed in Subject 2 and Subject 1 for IL‐6 and IL‐8, respectively, at T2 and T3 hours of measurement, the levels do not change significantly over time. Subject 3 demonstrated higher cytokine levels as compared to Subjects 1 and 2. While the exact reason for this is unknown, there may be several factors including dietary intake that may have influenced the levels and will require further investigation. Next, we wanted to understand if the levels of cytokines change over days. Cytokine levels for IL‐8 and IL‐10 were measured over 4 successive days (Figure S10D). The results demonstrate that cytokine levels remained <12 pg mL^−1^ and did not change significantly over the measured time period (*p* > 0.05). While this work demonstrates the proof‐of‐feasibility of wearable sweat sensing platform for continuous monitoring through affinity‐based mechanism, future efforts can be emphasized in implementing methods to regenerate the sensor surface of affinity‐based assays for enhancing the performance for continuous monitoring.^48^ However, we believe that these results provide a basis for future exploration on continuous monitoring of cytokines for various inflammatory/infectious diseases.

### 2.5 Clinical utility of SWEATSENSER for infection detection from eccrine sweat

**FIGURE 5:**
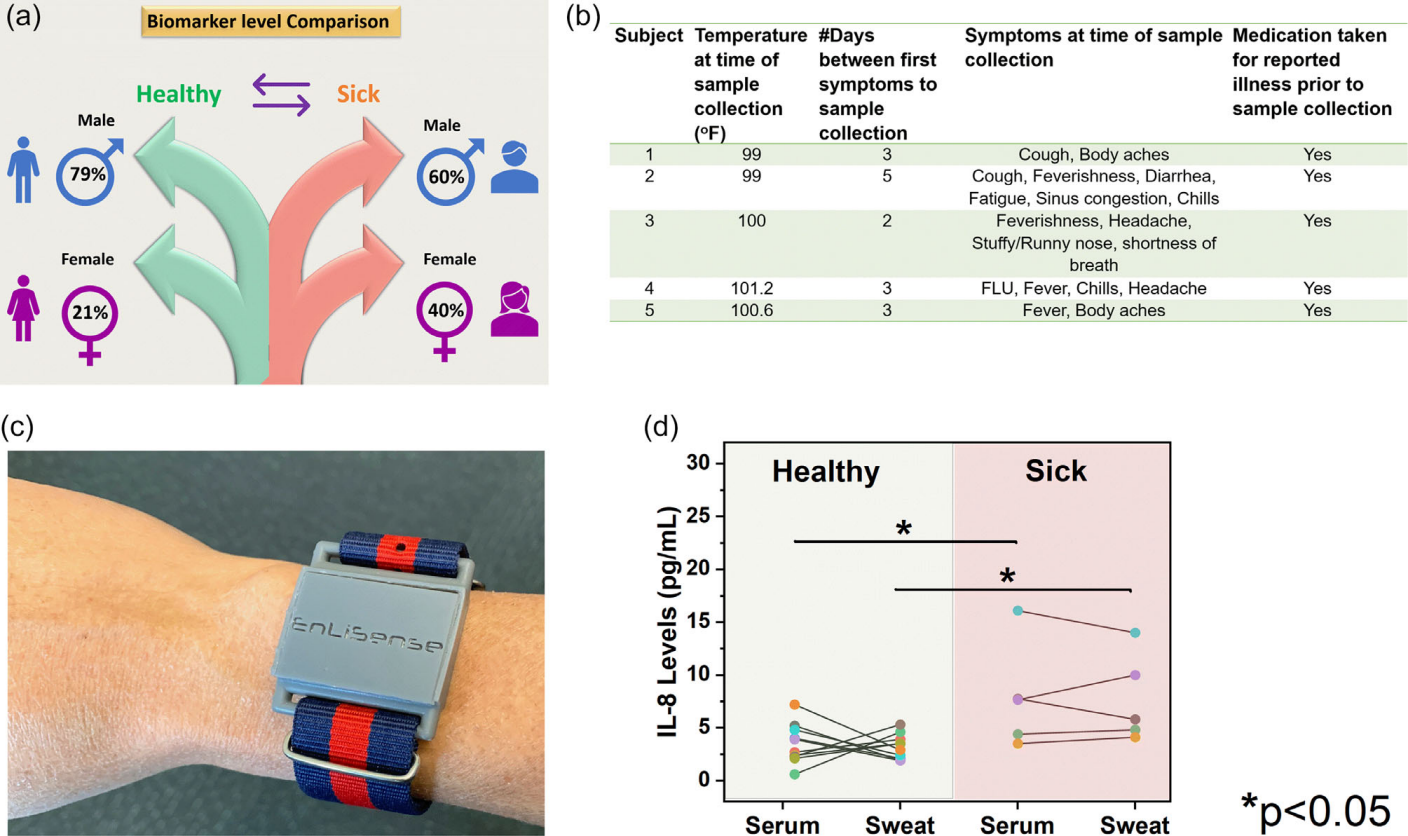
Serum to sweat IL‐8 levels in healthy and sick cohort. (a) Schematic of subjects recruited for serum to sweat correlation. (b) Details of vitals and illness of each individual in sick cohort. (c) SWEATSENSER device worn on hand by a subject recruited for the study. (d) Comparison of IL‐8 levels between healthy (*n* = 10) and sick cohort (*n* = 5). The levels between serum to sweat are also compared for each subject. Statistical significance *p* < 0.05 was achieved between healthy and sick cohort using ANOVA (*α* = 0.05) in both serum and sweat

The SWEATSENSER was then used to identify the levels in the sick cohort and compare to the serum levels to demonstrate the technology's feasibility for noninvasive monitoring of inflammatory response (Figure 5(a)). Ten healthy subjects and five sick subjects were recruited in compliance with the approved IRB protocol as described in Table S5 (Supporting Information). Among the five patients, three were male and two were female. All the subjects were taking medications. The description of sickness type of each patient has been described in Figure 5(b). A comparison of IL‐8 levels between healthy and sick cohort in passively expressed sweat was performed. Figure 5c shows the SWEATSENSER worn by a subject. While real‐time monitoring of infection‐related cytokine markers using SWEATSENSER can offer significant strides in tracking infection, it is also important to understand how sweat cytokine levels compare to its systemic circulation. Figure 5d shows a comparison of IL‐8 serum levels to SWEATSENSER measured IL‐8 levels from passively expressed sweat in healthy and sick cohort. Sweat IL‐8 levels are in the similar range as the serum IL‐8 levels of 2–15 pg mL^−1^. The slopes of serum to sweat levels follow a similar pattern among most healthy subjects with sweat levels lower than that of serum, thus showing a definitive relationship between serum to passively expressed sweat cytokines. An average sweat‐to‐serum ratio of IL‐8 ~ 1.01 from nine volunteers (one data point excluded as an outlier) was obtained. Furthermore, a similar relationship was observed in a patient cohort of five subjects. These results confirm that the levels of IL‐8 in sweat can be correlated to that in circulation. IL‐8 levels in patient cohort are relatively higher in serum and sweat compared to the healthy cohort. A statistical significance *p* < 0.05 using analysis of variance (ANOVA) (*α* = 0.05) for IL‐8 was determined between healthy and sick cohort for both serum and sweat, thus demonstrating that eccrine sweat may be used for determining infections. In the sick cohort, levels were comparable between female and male subjects. Four subjects were mildly sick with symptoms such as fever, cough, and chills, while one subject tested flu positive (Figure 5B). These results present some interesting perspectives and signify the role of noninvasive sweat‐based monitoring for diagnosis and prognosis of diseases. Subjects 2 and 5 demonstrated a similar kind of symptoms. Three subjects had fever at the time of measurement while two subjects did not have fever at the time of sample collection, but experienced other inflammatory or viral infection symptoms as described in Figure 5b. However, these two subjects also had fever on the previous days prior to sample collection. All the subjects had taken fever‐reducing medication. It should be noted that Subject 4 was sicker compared to other patients as this patient tested flu positive while others had minimal fever. This is also reflected in higher IL‐8 levels in serum and sweat, of Subject 4 than other subjects in the sick cohort. Interestingly, the levels in patient cohort were not extremely elevated and closer to the healthy cohort, although the levels of sick cohort are statistically significant from the healthy cohort. We hypothesize the relatively lower levels were probably because the samples (serum and sweat) were collected at least 2–3 days after the occurrence of illness. It could also be that these patients were taking medication and were in their recovery phase. However, the key takeaway from this study is that such nuanced changes in levels can be effectively captured by the SWEATSENSER to identify illness. Furthermore, the finding that the levels of sweat in healthy subjects is lower compared to the sick cohort demonstrates the significance of continuous monitoring of illness in real time. These preliminary results present an understanding on the relationship between systemic circulation levels of cytokines to that of circulation in passive eccrine sweat. Thus, setting the path for future studies to explore and validate serum to sweat relationship for enabling sweat analytics as a clinical diagnostic platform for noninvasive, robust, and pre‐symptomatic tracking of infection.

## 3 CONCLUSION

This work establishes a wearable technological platform that can detect infection/inflammation noninvasively from eccrine sweat. Such a technological platform can empower users with actionable data for necessary precautions even prior to experiencing infection symptoms. The holy grail of the developed SWEATSENSER technology is to directly measure host immune cytokine markers in passive eccrine sweat for presymptomatic infection tracking. This type of a real‐time monitoring system can offer a fundamental change in the approach of the way infections are being diagnosed, assessed, and treated. The developed wearable SWEATSENSER can be visualized to aid in presymptomatic reporting of infection and also for monitoring therapeutic treatment regimens through real‐time monitoring of cytokines that can mitigate severe consequences of pathogen attack. Additionally, the continuous monitoring of cytokines offers a new perspective in real‐time monitoring of sweat protein markers, which have thus far only been analyzed through cumbersome lab techniques. The ability demonstrated by the SWEATSENSER in differentiating biomarker levels between healthy and sick cohort in passively expressed eccrine sweat will offer new insights to clinicians for better management and prognosis of inflammatory/infectious diseases. The agreement between the developed device and state‐of‐the‐art reference method through preclinical healthy cohort studies establishes the usability of SWEATSENSER device for on‐field testing. SWEATSENSER design's novelty offers the flexibility to incorporate other protein biomarkers of interest based on the application needs to various other inflammatory disease monitoring and is therefore not restricted to these four cytokines. Although the current technology is affinity based and the sensor surface is not regenerated, incorporating methods to regenerate surface can further improve the longevity, lifespan, and functional capabilities of the sensor. However, the correlations established between serum to sweat IL‐8 levels and the ability of identifying illness using SWEATSENSER will be of significant interest to researchers, clinicians to look at sweat as an alternative biofluid for improving therapeutic diagnosis and prognosis of several inflammatory/infectious diseases. Future work will be focused on further optimization, improvisation, and strengthening the clinical validation of SWEATSENSER for acute respiratory infections, flu, and other viral/bacterial infections. Additionally, studies can be conducted to sample serum and sweat at multiple time points within a day and between days to better understand diurnal and circadian variations of cytokine biomarkers in sweat and their physiological role.

## 4 EXPERIMENTAL SECTION

### 4.1 Reagents and instrumentation

DTSSP (3,3′‐dithiobis[sulfosuccinimidyl propionate]) cross‐linker and phosphate‐buffered saline (PBS) were purchased from Thermo Fisher (USA). The capture probe monoclonal antibodies (mAb) and recombinant proteins for IL‐6, IL‐8, and TNF‐α were purchased from Abcam (MA) while IL‐10 antibody was purchased from Thermo Fisher. IL‐6 ELISA kit was procured from Abcam, TNF‐α from Thermo Fisher, and IL‐8 and IL‐10 kits were purchased from Raybiotech. The reagents for ELISA measurements were stored and reconstituted according to the protocol from the manufacturer.

### 4.2 Sensor immunoassay functionalization

10 mM DTSSP cross‐linker mixed with 10 μg mL^−1^ of monoclonal capture antibody of each marker immobilized on the sensing electrode surface via thiol‐binding mechanism. Each working electrode specific to a biomarker was independently functionalized to achieve specific detection of target biomarkers. For sensor performance metric studies such as repeatability, reproducibility, and stability, the functionalized sensors were lyophilized and immediately vacuum sealed until further use.

### 4.3 SWEATSENSER device

The SWEATSENSER device consists of a replaceable sweat sensing strip and an electronic reader. The sensing strip is functionalized with specific target capture probes, that is, IL‐6, IL‐8, IL‐10, and TNF‐α monoclonal antibodies as described previously. This sensor strip is mounted onto a wearable electronic reader that transduces the impedance response from the sensor and reports the measured biomarker levels in sweat. The sensor fabrication process has been adapted from Munje et al. and has been described in detail previously.^47^ Briefly, a sensing‐electrode system is fabricated on the SWEATSENSER strip for enabling transduction of affinity‐based interaction between the target biomarker and capture probe antibody into a measurable electrochemical signal. The sensor has been designed to handle low volumes of sweat and the design has been optimized by considering the sweat gland density, sweating rate, and surface area of contact. Non‐faradaic electrochemical impedance spectroscopy was used as the detection modality to determine the sensing response of the binding interactions between the specific capture probe and target molecule. A very low input of 10 mV sinusoidal voltage was applied to the sensing electrodes and the change in impedance due to the binding interaction between target molecule and capture probe antibody resulting in charge modulation was recorded at 180 Hz. Calibration curve for each study marker was developed by measuring the impedance response for varying concentrations of the target analyte over the physiological range of 0.2–200 pg mL^−1^.

### 4.4 SWEATSENSER electronic reader design

The architecture of the electronic reader used in this work is shown in Supporting Information Figure S11. A component off‐the‐shelf (COTS) temperature and RH sensor (Texas Instruments Inc.), a battery management system (Texas Instruments Inc.) and Bluetooth Low Energy (BLE) module (RayTac Corporation, Taiwan) were added to a central arm core processor. The integrated temperature and RH sensor help measure the temperature and RH conditions at the epidermal surface where the sensor is applied. The battery management system manages a regulated charge–discharge operation of the battery in the recommended safe operating region. The bluetooth low energy module helps establish wireless communication to a smartphone with the lowest power consumption and form factor. A 3.7 V LiPo battery with suitable charge capacity was used that has a lifetime of 168 h on single charge.

### 4.5 SWEATSENSER characterization

FTIR spectroscopy was performed using Nicolet iS‐50 (Thermo Scientific Inc.). The samples were prepared as previously described in SWEATSENSER design section and the measurement was done in attenuated total reflectance mode. Two‐hundred fifty‐six spectral scans at a resolution of 4 cm^−1^ were collected using Germanium crystal for a wavelength range between 4000 and 600 cm^−1^. Electrochemical characterization of antibody functionalization was performed using a Gamry Instruments potentiostat. A small input voltage of 10 mV AC was applied, and the impedance response was measured over a frequency range of 1 Hz to 1 MHz.

### 4.6 Human subjects sweat sample collection, handling, and processing

Sweat samples were collected, processed, and evaluated in compliance with the protocol approved by the Institutional Review Board (IRB) at the university (IRB# 19‐42). Twenty‐six human subjects were recruited with their informed written consent for participation in this study. A detailed description of overall information of enrolled subjects is described in Supporting Information Table S1. The protocol for sweat sample collection was adapted from Hladek et al.^38^ where two FDA‐approved PharmChek® patches were placed (one on each arm of the volunteer) at the same time. One patch was removed at 24 h and the other at 72 h from the time the patch was put on the volunteer. The samples were de‐identified and stored in freezer immediately upon collection in 5 mL tube until used for analysis. Samples were removed and thawed prior to analysis. 2 mL of elution buffer prepared as per the protocol described in Hladek et al. was added to the tube containing the sample. The sample with the elution buffer in the 5 mL tube was placed in a secondary container with ice and operated on a shaker plate for 45 min. Next, the sample tube was centrifuged at 20*g* for 3 min operated at 4°C. Any remaining sample was extracted by placing the patch in a syringe and the residual sample fluid was extracted by squeezing out that was entrapped in the patch.

### 4.7 ELISA analysis

The extracted patch samples were evaluated using ELISA as the reference method to establish correlations with SWEATSENSER. The individual kit's protocol was followed as per manufacturer instructions. Absorbance at 450 nm was used to read the OD response and determine the levels of biomarkers in samples.

### 4.8 In vitro sweat sensor analysis for patch samples

The extracted and aliquoted samples were evaluated and compared with the ELISA results to better understand the accuracy of the SWEATSENSER device in reporting the levels of biomarkers in human subjects. As the collected samples were from a healthy cohort, elevated concentrations were spiked into the buffer to demonstrate the performance of device in capturing the concentration levels of sick cohort. These concentrations were identically evaluated with ELISA as well. The measured levels using the SWEATSENSER device was compared with the obtained ELISA results using statistical methods such as Pearson's correlation and Bland–Altman analysis to determine the efficacy of the sweat sensor when compared to the reference ELISA method.

### 4.9 On‐body SWEATSENSER measurements on human subjects for comparing cytokine levels in healthy and sick cohort

All enrolled subjects with written informed consent wore the SWEATSENSER devices during the duration of the testing and measurements were recorded in compliance with the protocol approved by the IRB# 19‐136. Serum and sweat samples were collected from 10 healthy and 5 sick subjects to perform serum to sweat correlation in healthy and sick cohort. Prior to the sample collection, temperature was checked for all the subjects and a questionnaire was recorded on the patient symptoms. This was followed by collection of ~5 mL blood sample by a physician which was converted to serum and stored at ‐20° C until further use. Then, SWEATSENSER was immediately placed on the lower forearm of the subject for ~1 h to allow for sufficient sweat collection. SWEATSENSER device was covered with full‐sleeve or a sweat‐band to keep the device enclosed and prevent interactions with the ambient environment. The inclusion criterion for sick cohort were that subjects should have been experiencing inflammatory/infection symptoms such as fever, flu, or diagnosed with some type of bacterial or viral infection. Subjects were asked if they were taking any medication related to illness in the last 48 h. No dietary information was collected from the subjects. The collected serum levels were analyzed using a standard laboratory reference method. Analysis of on‐body sweat measurements using the SWEATSENSER were performed using the developed analytical algorithm. Complete details of the analytics for determining sweat levels through continuous monitoring has been described in Figure S9 (Supporting Information).

### 4.10 Statistical analyses

Several statistical tests were performed using Origin Pro. Statistical *t*‐test and ANOVA was carried out with a CI of 95% for studies such as reproducibility, on‐body temporal monitoring. Pearson's correlation and Bland–Altman analysis were performed to determine the extent of agreement of sweat sensor with the reference method.

## AUTHOR CONTRIBUTIONS

**Badrinath Jagannath:** Conceptualization; data curation; formal analysis; methodology; writing‐original draft; writing‐review & editing. **Kai‐Chun Lin:** Conceptualization; data curation; formal analysis; writing‐original draft; writing‐review & editing. **Madhavi Pali:** Conceptualization; data curation; formal analysis; writing‐review & editing. **Devangsingh Sankhala:** Data curation; formal analysis; writing‐original draft; writing‐review & editing. **Sriram Muthukumar:** Conceptualization; formal analysis; funding acquisition; supervision; writing‐original draft; writing‐review & editing. **Shalini Prasad:** Conceptualization; formal analysis; funding acquisition; supervision; writing‐original draft; writing‐review & editing.

## CONFLICT OF INTERESTS

Drs Shalini Prasad and Sriram Muthukumar have a significant interest in EnLiSense LLC, a company that may have a commercial interest in the results of this research and technology. The potential individual conflict of interest has been reviewed and managed by The University of Texas at Dallas, and played no role in the study design; in the collection, analysis, and interpretation of data; in the writing of the report, or in the decision to submit the report for publication. SWEATSENSER device and technology platform are a proprietary of EnLiSense LLC.

## Supporting information

**Appendix S1**: Supporting Information

## Data Availability

Research data are not shared.
