# Supplementary material for: Temporal profiling of cytokines in passively expressed sweat for detection of infection using wearable device
Source: Bioeng Transl Med. 2021 Apr 1;6(3):e10220. doi: 10.1002/btm2.10220 (PMC8459593; doi:10.1002/btm2.10220)
Supplement: Supplementary file 1 — Appendix S1: Supporting Information [file BTM2-6-e10220-s001.docx]

Supporting Information

**Temporal Profiling of Cytokines in Passively Expressed Sweat for Detection of Infection using Wearable Device**

*Badrinath Jagannath^1^, Kai-Chun Lin^1^, Madhavi Pali^1^, Devangsingh Sankhala^2^, Sriram Muthukumar^3^*, Shalini Prasad^1^**

1. Department of Bioengineering, University of Texas at Dallas, Richardson, TX, 75080, USA
2. Department of Electrical Engineering, University of Texas at Dallas, Richardson, TX, 75080, USA
3. EnLiSense LLC, 1813 Audubon Pond Way, Allen, TX, 75013, USA

* Corresponding Authors: sriramm@enlisense.com, [shalini.prasad@utdallas.edu](mailto:shalini.prasad@utdallas.edu)

**
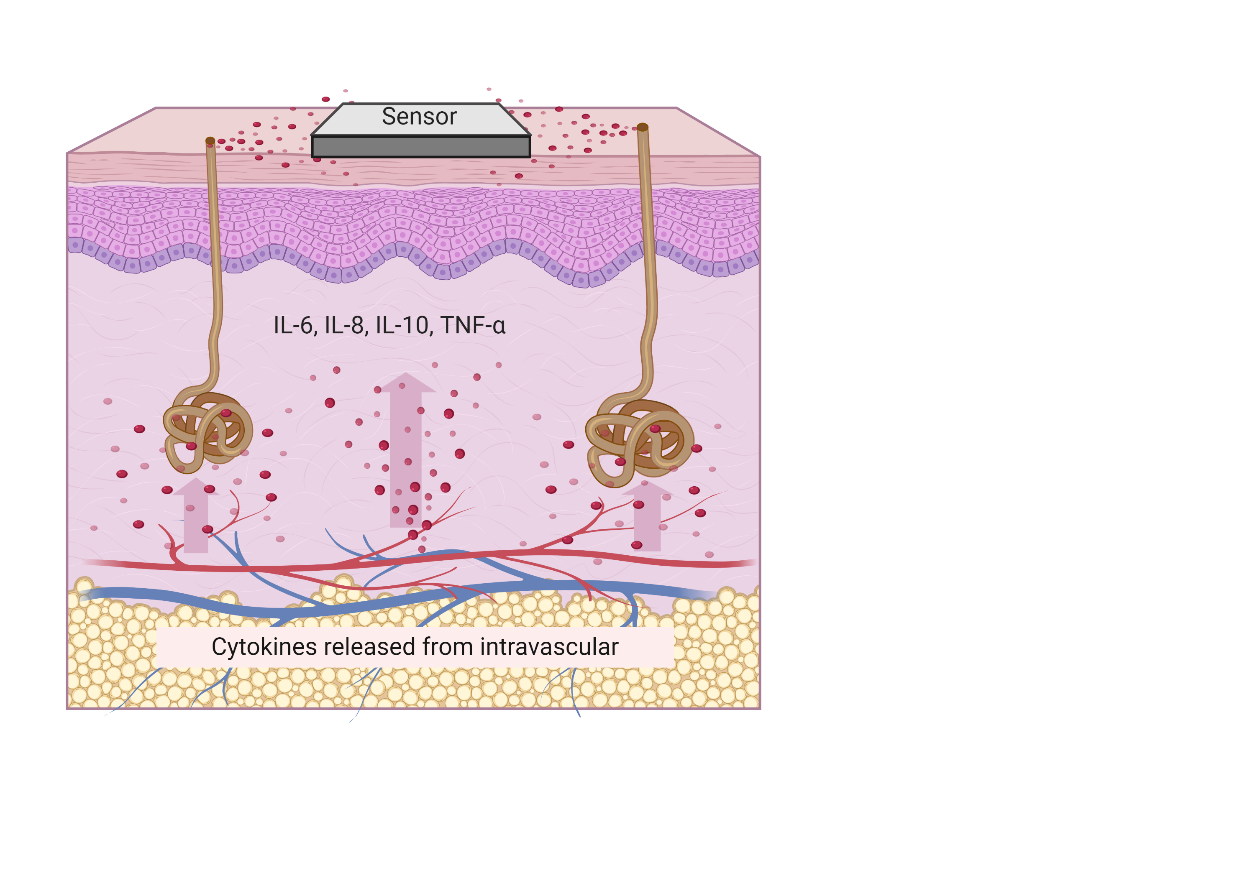
**

**Fig. S1:** Schematic representation of cytokine and protein production in circulation being diffused into the sweat pores due to the pathogen attacking cell. Note: Fig. was created in Biorender.

**A**

**
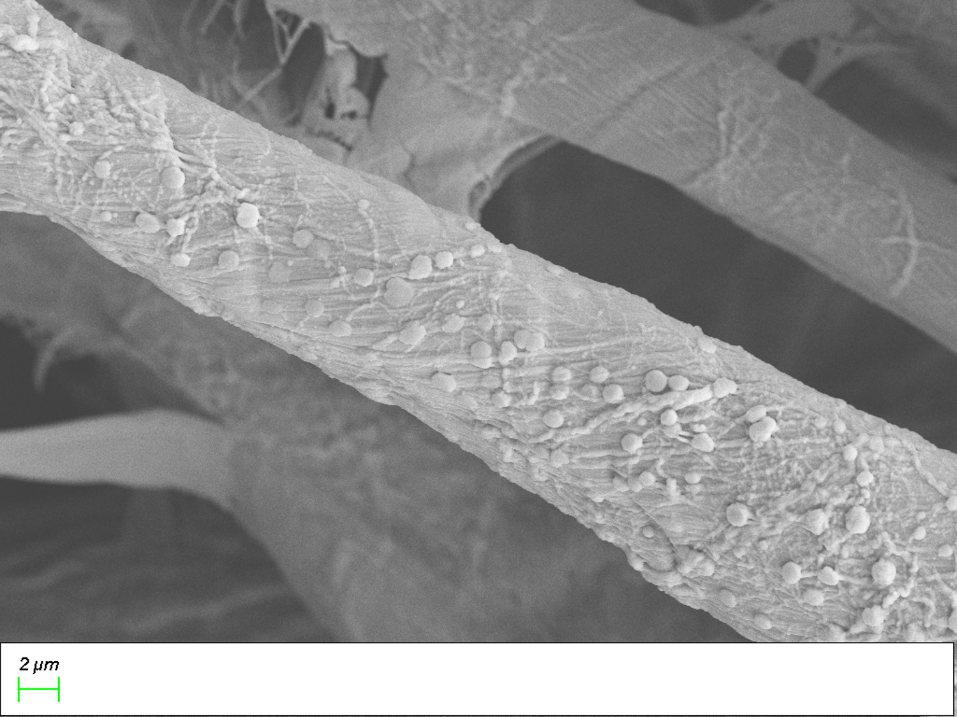
**

**B**


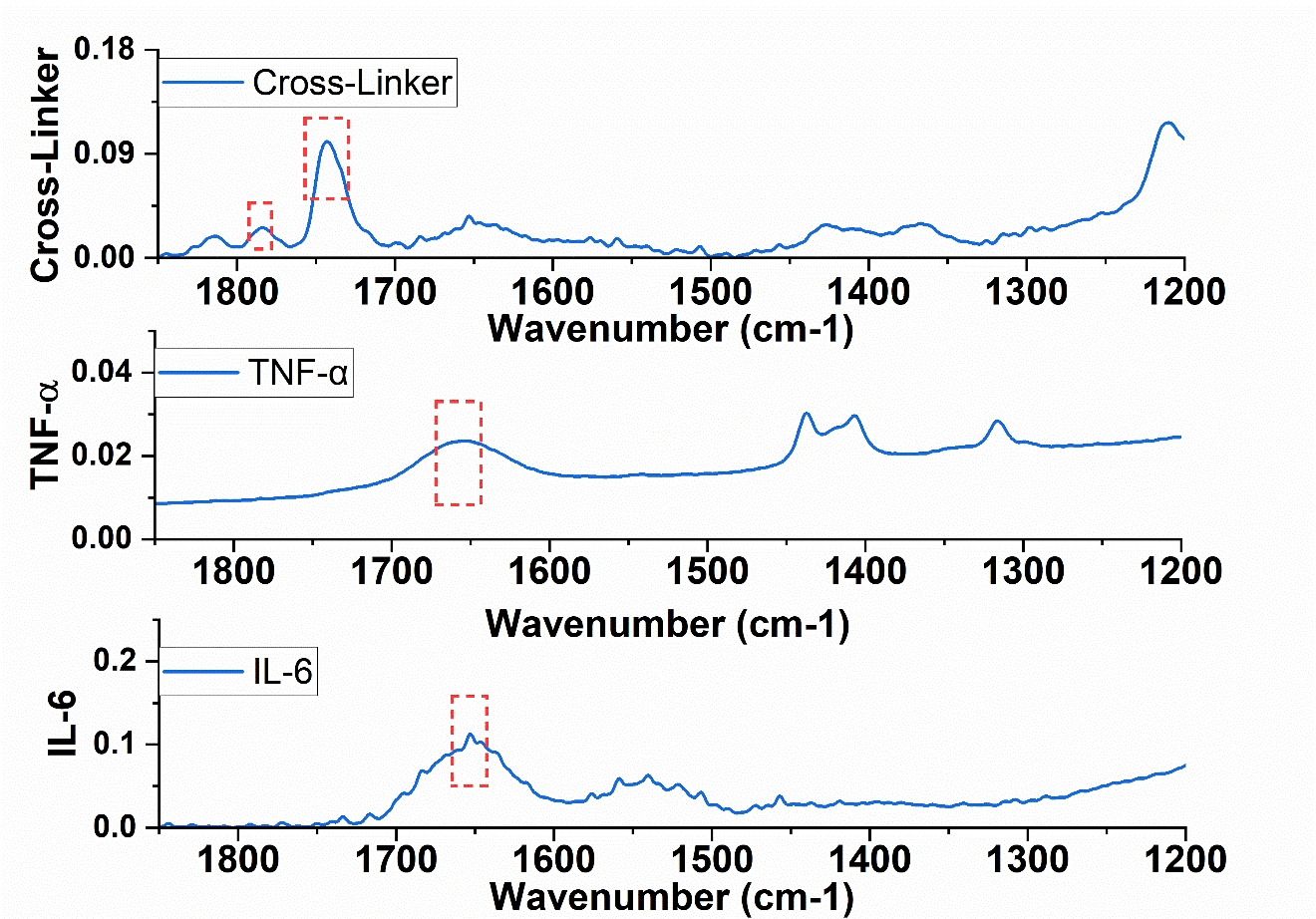


**Fig. S2:** (A) SEM image of uniform deposition of ZnO on patch membrane. (B) FT-IR spectra confirming conjugation of the antibodies on the sensor surface.

**

**

**Fig. S3:** (A) Nyquist plot of blank sensor prior to antibody functionalization. (B) Nyquist plots of IL-6, IL-8 and TNF-a antibody functionalized sensors. (C) Electrochemical equivalent circuit of electrode/solution interface. (D) Double layer capacitance computed using circuit fit for blank and antibody functionalized sensors.

**KD value interpretation:**

Y= Bmax/X+Kd (1)

X: Response

Y: Response/Concentration

Bmax: Maximum response

Kd: Equilibrium dissociation constant





**Fig. S4:** Nyquist plot with varying concentrations of target Anlayte (A) IL-6, (B) IL-8, (C) IL-10, (D) TNF-α.





**Fig. S5:** Precision of SWEATSENSER calcualted as %coefficient of variation (%CV) for normal and elevated doses which is less than the CLSI limits.


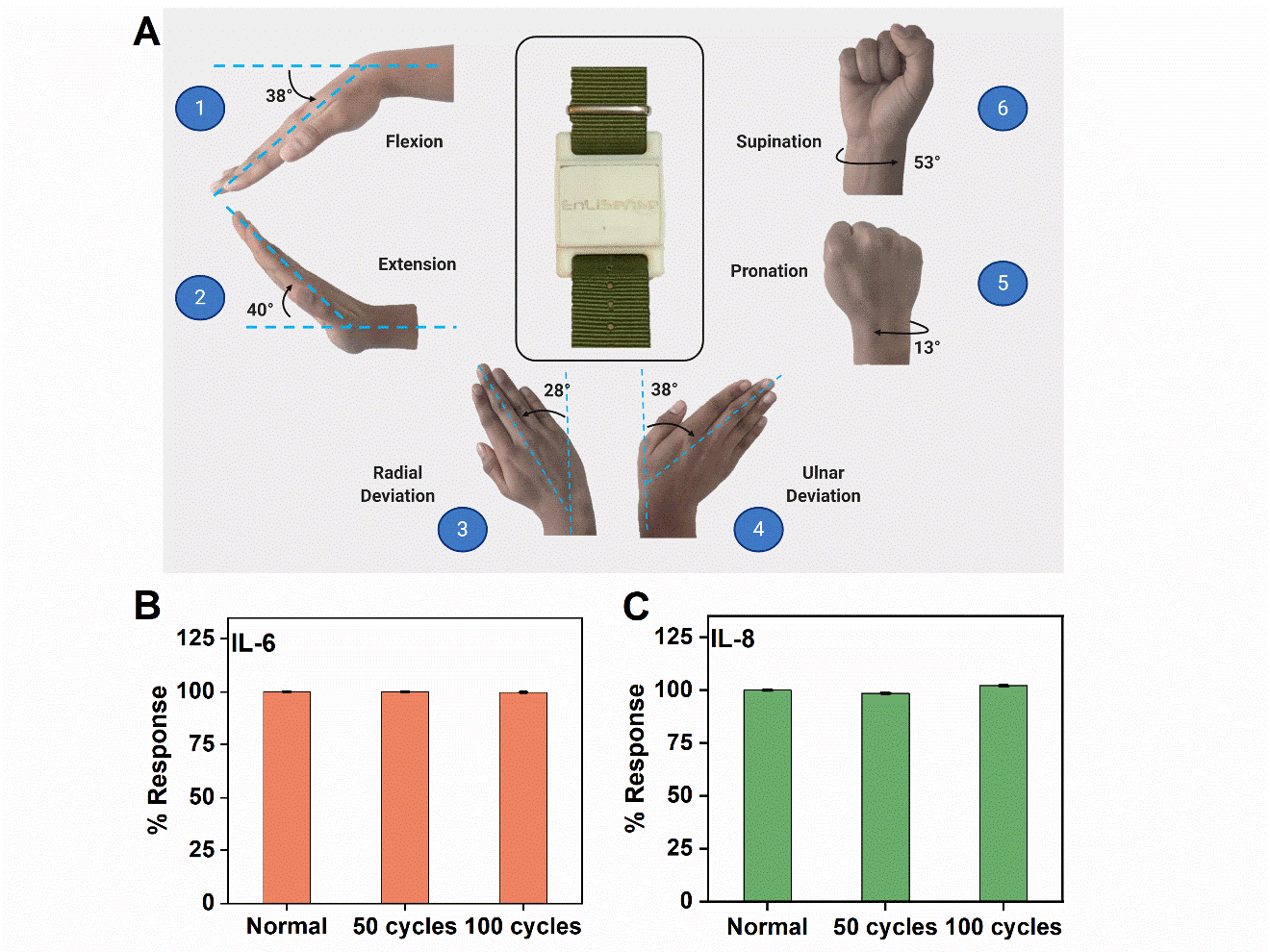


**Fig. S6:** Mechanical resiliency of SWEATSENSER. (A) The device was worn and subjected to various mechanical motions of hand as represented from steps 1-6. Steps 1-6 indicate 1 cycle. These steps were repeated upto 100 times and the response was recorded at 50 and 100 times. (B, C) The response of SWEATSENSER is retained for measured IL-6 and IL-8 even after 100 cycles of mechnical hand motion from step 1-6.

Table S1: Summary of Human subjects for sweat sample collection using patch

| **Human Subject Cohort Summary** | |
| --- | --- |
| # of Volunteer measurements | 26 |
| Age range | 18- 65 years |
| Gender | Male: 14; Female: 12 |
| Medication consumption | None |


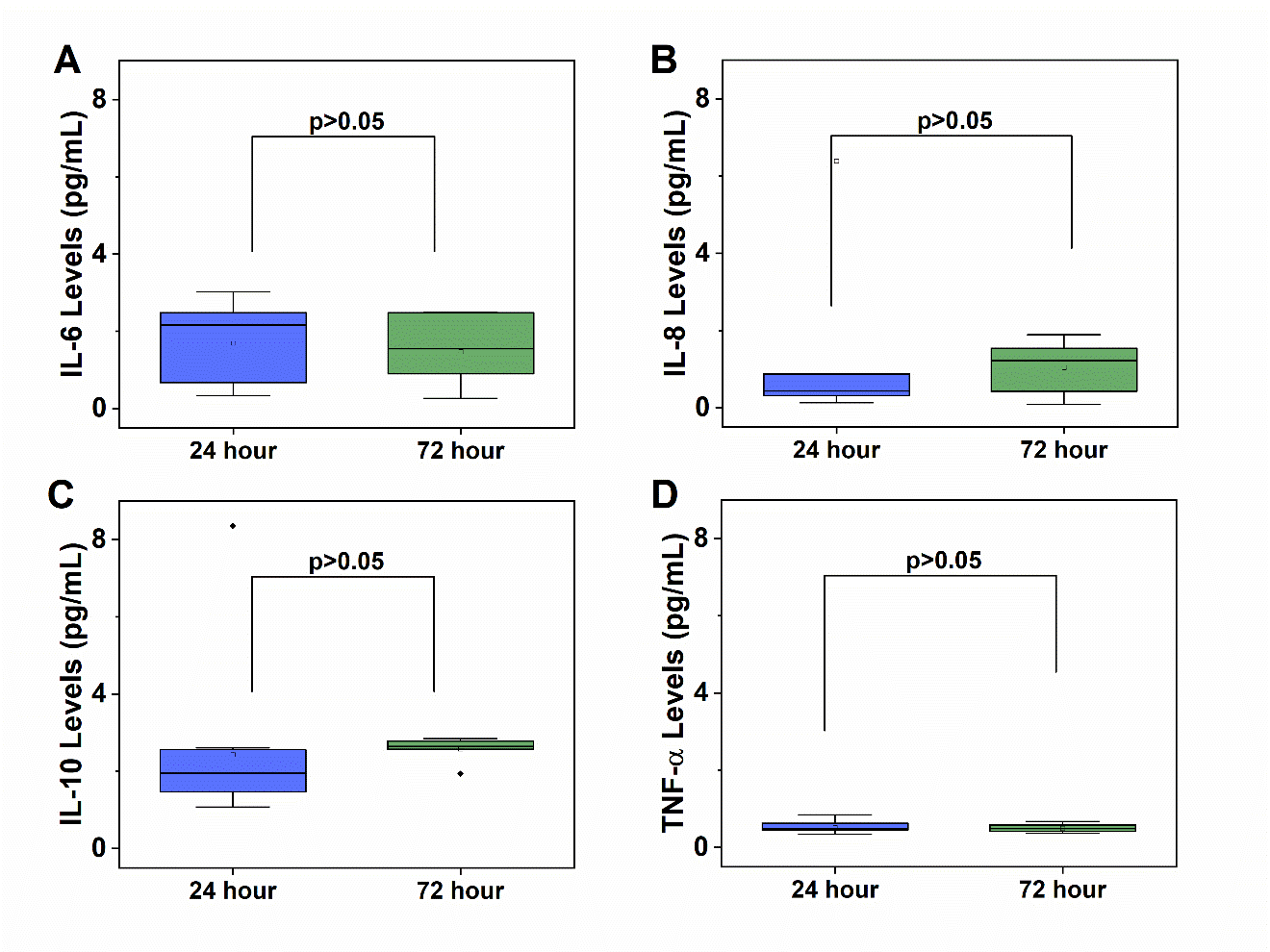


**Fig. S7:** Comparison of cytokine levels collected at 24 and 72 hours using PharmChek patch from healthy cohort measured using ELISA. The box ploys indicate that levels in healthy cohort does not vary between 24 hours and 72 hours, with a statistical insignificance of p>0.05.

Table S2: Comparison of study marker levels between SWEATSENSER and reference ELISA from healthy subject cohort

| Study Biomarker | Mean Bias  (pg/mL) | 95% C.I (±1.6SD)  (pg/mL) | Pearson’s coefficient ( r ) |
| --- | --- | --- | --- |
| IL-6 | -1.99 | -16 - 12 | 0.99 |
| IL-8 | -9.43 | -22 - 3.86 | 0.99 |
| IL-10 | -0.5 | -8.1 - 7.18 | 0.99 |
| TNF-α | 0.55 | -3.72- 2.61 | 0.99 |

Table S3: Levels of pro and anti-inflammatory cytokines in passive and stimulated eccrine sweat

|  | IL-6  (pg/mL) | IL-8  (pg/mL) | IL-10  (pg/mL) | TNF-α  (pg/mL) |
| --- | --- | --- | --- | --- |
| Passive sweat | 0.26 - 3 | 0.08 - 17 | 1- 8.3 | 0.3- 0.9 |
| Stimulated sweat | Not reported | 0.9- 3.25 | 0.4- 2.75 | Not reported |





**Fig. S8:** **A.** Temperature measured in three healthy subjects across varying time points. **B.** Monitoring of perspiration using realtive humidity (%RH) sensor for three healthy subjects measured at various time points.


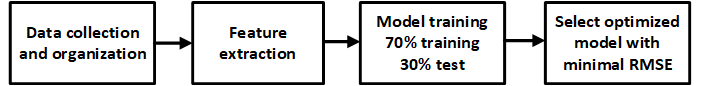


**Fig. S9:** Schematic of the methodology implemented for training and prediction of levels of the biomarkers. Feature extraction of IL-8 and the associated metrics.

Fig. S9 shows the training architecture of the proposed training system. This training system consists of four major parts: (1) Data collection and organization (2) Feature selection and extraction (3) Model training (4) Model optimization for low error. The training data obtained from multiple sensors is collected and collated. This training data was generated by simulating a dose profile within physiological range. Once the data is collected, it is split into multiple model inputs or features, which are closely related with the operation of the sensor. Normalized impedance is used as an input as it helps confine the data to a normal distribution with mean of 0 and standard deviation of 1 ^[1]^ which helps in reducing training the model to perform more efficiently. Additional features are also added to satisfy the time-dependent response change of the sensor. The running difference of normalized impedance helps to define the rate of change of sensor impedance in a short period of time. The percentage change of normalized impedance defines the change of sensor impedance from the time of start of use for the sensor. These features account for the cumulative accumulation of doses on the sensor.

The training was performed on N = 20 by splitting the collected data into groups of train series and test series in a 70:30 splits. Various commonly known training models such as linear regression, quadratic support vector machine (SVM), bagged ensemble regression and decision tree regression were studied for this application. Table S5 shows the highest R^2^ for the decision ensemble regression system. A mean of the normal distribution of error centered close to 0 pg/mL with a span of +/- 6 pg/mL was achieved.

Table S4: IL8 training outcomes

| **Model type** | **R^2^** |
| --- | --- |
| Linear regression | .56 |
| Tree | 0.96 |
| **Ensemble** | **.98** |
| Quad SVM | .47 |





**Fig. S10:** On-body continuous monitoring of cytokines. (A, B, C) SWEATSENSER demonstrates levels of biomarkers in 3 subjects measured every 1 hour. (D) Box-plot of IL-8 and IL-10 in multiple healthy volunteers measured across various time-points in a day demonstrating there is no significant change in cytokine levels over time in healthy cohort.

| **Human Subject On-body Testing** | |
| --- | --- |
| # of Volunteers Measurements | 15 |
| Age range | 18- 65 years |
| Healthy Cohort | Male: 7; Female: 3 |
| Sick Cohort | Male: 2; Female: 3 |

Table S5: Summary of Human subjects for healthy to sick cohort comparison


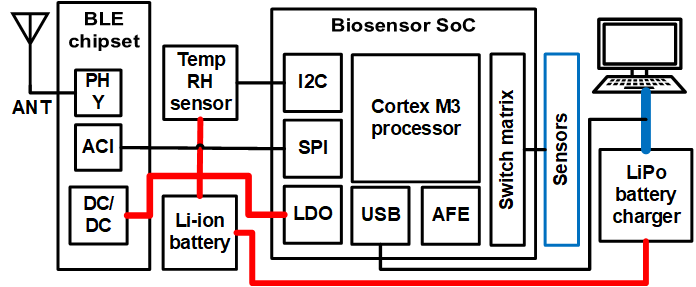


**Fig. S11:** Architecture of the SWEATSENSER electronic reader indicating various components used for transduction and processing of the biochemical interaction.

1. M. Shanker, M.Y. Hu, M.S. Hung, *Omega.* 1996, **24**, 385–397.
